# The impact of the embryo banking on the cumulative live birth rate in women with poor ovarian response according to the Bologna criteria

**DOI:** 10.1002/rmb2.12533

**Published:** 2023-08-26

**Authors:** Qiu Lin Ge, Xiao Ming Teng, Miao Xin Chen, Kun Ming Li, Ernest Hung Yu Ng, Zhi Qin Chen

**Affiliations:** ^1^ Center of Assisted Reproduction, Shanghai First Maternity and Infant Hospital Tongji University School of Medicine Shanghai China; ^2^ Department of Obstetrics & Gynaecology, School of Clinical Medicine, LKS Faculty of Medicine The University of Hong Kong, Hong Kong Special Administrative Region Hong Kong China

**Keywords:** cumulative live birth rate, embryo banking, IVF, POR, time to live birth

## Abstract

**Purpose:**

To evaluate the impact of embryo banking on the cumulative live birth rate (CLBR) and the time to live birth (TTLB) in poor ovarian responders (POR) according to the Bologna criteria.

**Methods:**

A total of 276 infertile women undergoing IVF with POR were included in this retrospective study. They were divided into two groups with (*n* = 121) or without (*n* = 155) embryo banking at the discretion of the attending physicians. A total of 656 and 405 stimulation cycles were started in the two groups respectively during the 24 month follow‐up.

**Results:**

The biochemical pregnancy, clinical pregnancy, ongoing pregnancy, and live birth rate per transfer were comparable between two groups (*p* > 0.05). The CLBR was significantly lower in the banking group than in the non‐banking group (31.4% (38/121) and 43.2% (67/151), *p* < 0.05). TTLB was significantly longer in the banking group (20.5 months vs. 16.0 months, *p* < 0.001). In the Kaplan–Meier analysis, the cumulative incidence of live birth was significantly lower in the banking group compared with the non‐banking group (Log rank test, chi‐square = 21.958, *p* < 0.001).

**Conclusions:**

Embryo banking in women undergoing IVF with POR based on the Bologna criteria reduces CLBR and lengthens TTLB when compared with no embryo banking.

## Graphical abstract

A retrospective cohort study of 276 infertile women undergoing IVF with poor response according to the Bologna criteria revealed embryo banking reduces the cumulative live birth rate and lengthens the time to live birth.

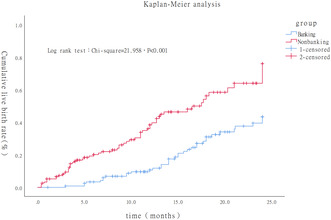

## 1 INTRODUCTION

Optimizing the outcome of in vitro fertilization (IVF) in women with poor ovarian response (POR) as defined by the Bologna criteria^1^ remains a challenge. Approximately 20% of all women undergoing IVF show POR to ovarian stimulation,^2^ which leads to lesser oocyte obtained, no good quality embryos for transfer, higher drop‐out rates^3^ and consequently results in lower live birth rates as compared to women with a normal ovarian response.^4^, ^5^, ^6^ Many strategies have been proposed to improve the treatment outcomes of POR patients; however, no single intervention or modification of the treatment protocol has been proved to be useful.^7^, ^8^, ^9^

Previously studies have shown that an increased gonadotrophin dose does not increase the number of oocytes/embryos and pregnancy rates with a single IVF cycle in predicted poor responders.^10^, ^11^, ^12^, ^13^ However, increasing the oocyte/embryo yield from consecutive stimulation cycles may be a new approach to overcome poor outcomes of POR.^14^, ^15^, ^16^, ^17^ The rationale behind this approach is that the IVF success rate could be improved if the number of available embryos for transfer is increased, mimicking a “normo‐responder” status. Regarding oocyte banking, published data demonstrated that this could be an effective approach towards improving IVF outcomes in POR patients.^14^, ^15^, ^16^ These data suggest that oocyte banking by vitrification for POR significantly reduces patients' drop‐out rates and IVF cancellation rates, as well as significantly increasing the live birth rate per intention‐to‐treat patient.^15^

In a more recent retrospective study, Datta et al.^18^ proposed embryo banking through multiple modified natural cycles that might give the opportunity of better selection and transfer of multiple embryos, leading to improved pregnancy outcomes in POR. Adopting this strategy, the vast majority of women with three repeated cycles may have at least one embryo for transfer, overcoming the high risk of cycle cancellation associated with a single natural cycle.^18^

At present, there are still no robust data indicating the efficacy of the “banking scenario” in women with POR who are undergoing IVF after multiple treatment cycles, when compared to the no embryo banking approach. Thus, the aim of this retrospective study was to compare the cumulative live birth rate and time to live birth (TTLB) between women undergoing IVF with POR according to the Bologna criteria in a 24 month follow up who had either embryo banking or no embryo banking. We hypothesized that embryo banking improved the cumulative live birth rate in women with POR.

## 2 MATERIALS AND METHODS

### 2.1 Participants

The study protocol was approved by the Institutional Review Board of the hospital (No. KS20163). Informed consent was waived because only unidentified data were used in this retrospective study.

Infertile women who underwent IVF in our center between October 2016 and March 2020 were included for this retrospective study if they had at least two of the following three features according to the Bologna criteria: (i) advanced maternal age (≥40 years) or any other risk factors for POR (such as previous ovarian surgery, genetic defects, chemotherapy or autoimmune disorder); (ii) a previous POR (3 oocytes with a conventional stimulation protocol); and (iii) an abnormal ovarian reserve test (antral follicle count (AFC) less than 5–7 follicles and serum anti‐Mullerian hormone (AMH) less than 0.5–1.1 ng/mL). Women were excluded if they were: (i) aged ≥46 years, (ii) having an abnormal uterine cavity shown on hysterosalpingogram or hysteroscopy, (iii) having moderate or severe endometriosis, (iv) using donor eggs/sperm, (v) undergoing pre‐implantation genetic testing, (vi) on adjuvant therapy, such as dehydroepiandrosterone and growth hormone. Each patient were followed up for 24 months from the first day of ovarian stimulation in the first IVF cycle.

Women were offered either embryo banking or no banking at the discretion of the attending clinicians or subject to the wishes of the couple after extensive counseling. In the banking group, all couples were offered several consecutive stimulation cycles until at least five frozen embryos were banked prior to embryo transfer (the number needed to reach a 52% cumulative live birth rate in a standard situation for normo‐responders.^19^ Frozen embryos were transferred afterwards until successful pregnancy or all embryos were utilized. Those couples who opted have fresh embryo transfer constituted the non‐banking group for comparison and they would use up their remaining cryopreserved embryos before starting another stimulation cycle.

In both groups either one or two frozen embryos were transferred, according to the patient's preference. Intervals between IVF cycles were determined by logistic reasons and patients' preference. All treatment cycles were performed within 24 months after the first day of ovarian stimulation in the first IVF cycle.

### 2.2 Ovarian stimulation

Women were started ovarian stimulation using either GnRH short/long agonist, GnRH antagonist protocol, or progestin‐primed ovarian stimulation (PPOS), mild stimulation and natural cycle at the discretion of the attending clinicians. For the long protocol, 1.25 mg GnRH agonist (Triptorelin acetate, Diphereline, Ipsen Pharma Biotech, France) was given for pituitary desensitization from the mid‐luteal phase in the previous cycle. On Day 2–3 of the menstrual cycle they underwent transvaginal ultrasound examination and serum estradiol measurement. hMG (Lebaode, Lizhu, China) or FSH (Puregon, Organon, Dublin, Ireland or Gonal F, Merck Serono S.p.A, Modugno, Italy) was given at 225–300 IU per day based on the antral follicle count (AFC), age of the woman, body mass index (BMI) and previous ovarian response, according to the standard operating procedures of the center. Ovarian response was monitored by serial transvaginal scanning with or without hormonal monitoring. Further dosage adjustments were based on the ovarian response at the discretion of the clinicians in charge. For the antagonist protocol, antagonist 0.25 mg daily (Cetrotide, Merck‐Serono, Fareva Pau, France) was given from the sixth day of ovarian stimulation until the day of ovulation trigger. For the PPOS protocol, medroxyprogesterone (MPA, 10 mg/d, Shanghai Xinyi Pharmaceutical Co., China) was given on the same day together with hMG or FSH stimulation. Mild stimulation was used by given clomifene citrate 100 mg for 5 days followed by hMG 150 IU per day until the day of ovulation trigger.

When 1–3 leading follicles reached ≥18 mm in diameter, triptorelin (0.1 mg; Decapeptyl, Ferring Pharmaceuticals, Netherlands) and hCG (2000 IU; Lizhu Pharmaceutical Trading Co., China) or Ovidrel 250 microgram (Merck Serono S.p.A., Modugno, Italy) were given to trigger final maturation of oocytes. Oocyte retrieval was performed around 36 hours following the ovulatory trigger.

### 2.3 Fertilization and embryo evaluation

About 2 h after oocyte retrieval, each oocyte was inseminated with approximately 20 000–30 000 motile spermatozoa. If the total number of motile sperm was <10^5^ after washing or normal morphology was <1%, intracytoplasmic sperm injection (ICSI) was performed. Oocytes were decoronated and checked for the presence of two pronuclei to confirm fertilization. Embryos were graded on day 3 after retrieval as grade one to grade six according to the evenness of each blastomere and the percentage of fragmentation.^20^ Embryos of 6–8 cells and of grade one or two were regarded as top‐quality embryos. Some non‐top‐quality embryos were placed in extended culture until they reached the blastocyst stage.

### 2.4 Fresh and frozen embryo transfer

In the non‐banking group, 1–2 fresh embryos were transferred on Day 3 after retrieval under transabdominal ultrasound guidance. Surplus day 3 embryos or day 5 or 6 blastocysts in the non‐banking group and all viable embryos/blastocysts in the banking group were vitrified. Those who did not get pregnant in the stimulated IVF cycle and those who postponed fresh transfer would undergo FET at least 2 months after the stimulated cycle. The details of vitrification and warming procedure are described elsewhere.^21^ After warming, embryos were transferred to a culture dish for evaluation and further embryo development. Only embryos with more than 50% of blastomeres present after thawing were transferred in frozen embryo transfer. Frozen embryo transfer was carried out in natural cycles for ovulatory women and in clomiphene induced or hormonal cycles for anovulatory women. The best quality embryos were replaced first in the banking group. Up to two embryos or blastocysts were transferred in frozen embryo transfers.

### 2.5 Outcomes measures

The primary outcome measure was the cumulative live birth rate (CLBR) of which the ongoing status had to be achieved within 24 months since the first day of ovarian stimulation in the first stimulation cycle. In addition, LBR was calculated by including the first live birth generated during the 24 months period whether via fresh or FET cycles. Secondary outcome measures included time to live birth measured as the time from the first day of ovarian stimulation in the first cycle to a live birth, number of stimulation cycles per woman, total number of oocytes obtained per woman, number of cycles canceled before oocyte retrieval, number of patients with no embryo for transfer, number of started cycle with no embryo for transfer, number of transfer cycles in both group; number of oocytes obtained, fertilized per retrieval, number of transferable embryos per retrieval, and total number of transferable embryos per woman. Clinical pregnancy, biochemical pregnancy (defined as a positive pregnancy test), ongoing pregnancy, miscarriage, multiple pregnancy, and implantation rates per transfer in both fresh and FET cycles, time to ongoing pregnancy/live birth were also compared.

A baby born alive after 22 weeks gestation was classified as a live birth. Clinical pregnancy was defined as the presence of at least one gestational sac on ultrasound at 6 weeks. Ongoing pregnancy was the presence of at least one fetus with heart pulsation on ultrasound beyond 10 weeks. Implantation rate was calculated as the number of gestational sacs seen on scanning divided by the number of embryos replaced. Miscarriage rate was defined as the number of miscarriages before 22 weeks divided by the number of women with clinical pregnancy. Cancellation rate was defined as number of patients with no viable embryo to transfer divided by number of patients that started ovarian stimulation.

### 2.6 Statistical analyses

Continuous variables are given as the mean ± SD if normally distributed, and as the median + interquartile range if not normally distributed. Statistical comparison was carried out by Student's *t*‐test, Mann–Whitney *U* test for continuous variables and chi‐square test for categorical variables, where appropriate. The Kaplan–Meier (KM) method was used to calculate the cumulative proportion of ongoing pregnancies leading to live births, and time to pregnancy was graphically depicted by cumulative incidence curves. The log‐rank test was used to measure whether significant differences existed in the cumulative incidence curves. Censored data were considered when patients did not return for treatment. Women who did not reach the primary outcome (live birth) including those achieving a continuing pregnancy that did not lead to live birth were also censored. A Cox proportional hazard model was used to evaluate the relative prognostic significance of independent variables in relation to CLBR.

Statistical analyses were performed using the Statistical Program for Social Sciences (SPSS Inc., Version 24.0, Chicago, USA). The two‐tailed value of *p* < 0.05 was considered statistically significant.

## 3 RESULTS

**FIGURE 1:**
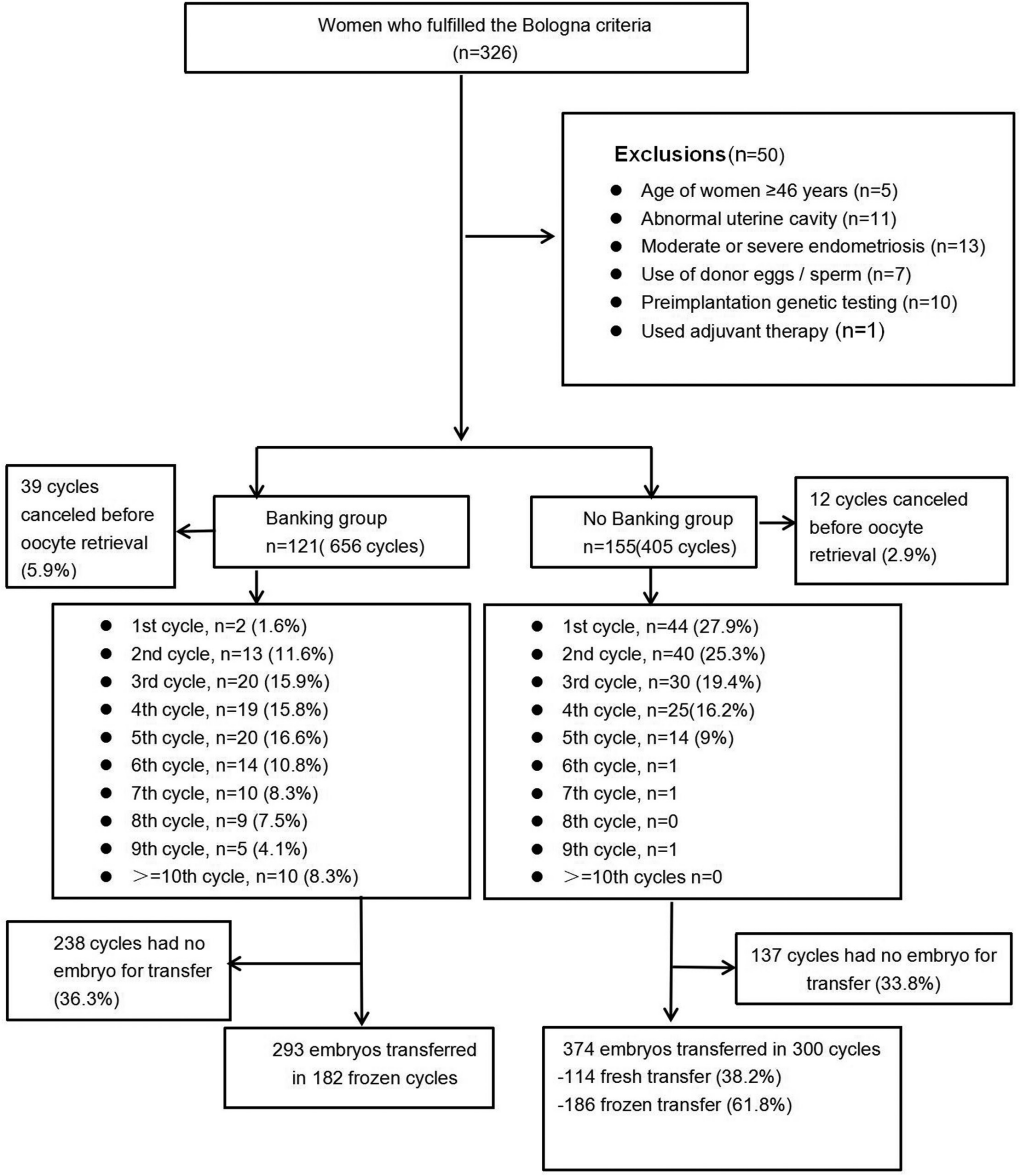
Patient flowchart.

A total of 326 women were screened, out of 276 women who met the selection criteria, 121 women had embryo banking while 155 women had no embryo banking. A total of 656 and 405 stimulation cycles were started in the banking and non‐banking group respectively within 24 months follow up (Figure 1).

### 3.1 Baseline characteristics (Table 1)

**TABLE 1:** Comparison of baseline characteristics between banking and non‐banking groups.

	Banking group (*n* = 121)	Non‐banking group (*n* = 155)
Age of women (years)	39 (36–41)	39 (36–40)
Infertility duration (years)	3 (1–5)	3 (1–5)
Body mass index (Kg/m^2^)	22 (19.8–23.7)	22 (19.8–24.7)
Primary infertility *n* (%)	47 (39.1)	73 (48.3)
Number of cycles, *n*	3 (1–5)*	2 (1–4)***
Basal antral follicle count	4 (1.8–6.8)	4 (2.0–6.5)
Basal FSH level (IU/L)	10 (7.5–13)	10.2 (8–13)
Serum AMH level (ng/mL)	0.68 (0.3–1.02)*	0.47 (0.24–0.96)**
Cause of infertility (%)		
Tubal	70 (57.9)	74 (47.7)
Male factor	15 (12.4)	18 (11.6)
Anovulatory	16 (13.2)	27 (17.5)
Unexplained	2 (1.7)	3 (1.9)
Mixed factors	18 (14.8)	33 (21.3)
Stimulation protocol (%)	*n* = 656	*n* = 405
Long agonist	48 (7.3)	28 (6.9)
Antagonist	47 (7.2)*	180 (44.4)***
Progestin‐primed ovarian stimulation	185 (28.2)*	56 (13.8)***
Mild stimulation	240 (36.6)*	68 (16.9)***
Short agonist	63 (9.6)	33 (8.1)
Natural cycle	73 (11.1)	40 (9.9)

*Note*: Values are median (25th and 75th percentile) or number (%).

* versus **p < 0.05, * versus ***p < 0.01.

The baseline characteristics are presented in Table 1. No significant differences were found in age of women, infertility duration, primary infertility, AFC, basal FSH, and cause of infertility, as well as BMI except for cycle number, and AMH. Stimulation protocol used in each group were different with more patients using PPOS and mild stimulation protocol in the banking group, while more used the antagonist protocol in the non‐banking group.

### 3.2 Comparison of stimulation cycle and embryological characteristics (Table 2)

**TABLE 2:** Comparison of cycle stimulation characteristics between banking and non‐banking groups.

	Banking group (*n* = 121)	Non‐banking group (*n* = 155)	
Total number of stimulation cycles	656	405	
No. of stimulation cycles per woman	5.3 ± 2.8*	2.6 ± 1.5***	
No. of cycles canceled before retrieval % (*n*)	5.9 (39/656)*	2.9 (12/405)**	
No. of cycles with no embryo for transfer % (*n*)	36.3 (238/656)	33.8 (137/405)	
Number of patient with no embryo for transfer % (*n*)	0/121	1/155	
Total FSH used (IU)	1200 (600–1800)*	1575 (750–2100)***	
Starting FSH dose (IU)	150 (150–225)*	225 (150–225)***	
Total FSH duration (days)	7 (5–9)*	8 (5–9)***	
Serum estradiol level on trigger day (pg/mL)	668 (338–1144)	660 (345–1002)	
Serum LH level on trigger day (IU/L)	4.3 (2–7)	3.7 (1.5–6.4)	
Serum progesterone level on trigger day (ng/mL)	0.89 (0.61–1.12)	0.79 (0.52–1)	
Maximal endometrial thickness (mm)	7.7 (5.0–10.0)*	8.0 (6.0–10.0)**	
Total no. of oocytes obtained	1402	824	
No. of oocytes obtained per woman	10 (8–14.8)*	4 (3–8)***	
No. of oocytes per retrieval	2 (1–3)	2 (1–3)	
No. of oocytes fertilized per retrieval	2 (1–3)	2 (1–3)	
No. of cleaving embryos per retrieval	1 (1–2)	1 (1–2)	
No. of top quality embryos per retrieval	1 (0–1)	1 (0–1)	
No. of transferable embryos per retrieval	1 (0–2)	1 (0–2)	
No. of transferable embryos per woman	5 (4–6)*	2 (1–4)***	
No. of patient with no transferable embryo, *n* (%)	0	1	

*Note*: Values are mean ± SD or median (25th and 75th percentile) or number (%).

* versus **p < 0.05, * versus ***p < 0.01.

The banking group had significantly higher number of stimulation cycles per woman and more cycles canceled prior to oocyte retrieval. The cancellation rate resulting no embryo for transfer was similar between the two groups. All patients in the banking group had embryo for transfer while only one patient in the non‐banking group had no embryo for transfer.

The banking group had significantly lower starting FSH dose, lower total FSH dose, lesser FSH stimulation days, lower endometrial thickness than the non‐banking group. Serum estradiol, LH and progesterone levels on the trigger day were comparable between the two groups.

The banking group had a significantly higher number of oocytes obtained per woman and total number of transferable embryos per woman than the non‐banking group. No differences were found in the number of oocytes obtained/fertilized per retrieval, the number of cleaving embryos, top quality embryos and transferable embryos per retrieval were found the two groups.

### 3.3 Pregnancy outcomes (Tables 3 and 4 and Figure 2)

**TABLE 3:** Comparison of pregnancy outcomes between banking and non‐banking groups.

	Banking group (*n* = 121)	Non‐banking group (*n* = 155)	Risk ratio (95%CI)
Patients (*n*)	121	155	/
Total number of embryos transferred	293	374	/
Total number of transfer cycles	182	300	/
No. of transfer cycle per women	1.5 ± 0.7*	1.9 ± 0.6**	
Cycle type of embryo transferred, *n* (%)			
Fresh transfer	0 (0)*	114 (38.2)***	/
FET	182 (100)*	186 (61.8)***	/
No. of embryos transferred, *n* (%)			
1	71 (39)*	227 (75.7)***	
2	111 (61)*	73 (24.3)***	
Stage of embryo transferred, *n* (%)			
Day 2/3 embryo transfer	172 (94.5)	28 (94.7)	
Blastocyst transfer	10 (5.5)	16 (5.3)	
Endometrial preparation, *n* (%)			
Natural cycles	51 (28)	54 (29)	/
Clomid‐induced	21 (11.5)	15 (8.1)	/
Hormonal cycles	110 (60.4)	117 (62.9.)	/
Endometrial thickness (mm)	9.7 (8–11)	10 (8–11.2)	/
Pregnancy outcome, *n* (%)			
Biochemical pregnancy (rate) per transfer	57/182 (31.3)	110/300 (36.7)	0.854 (0.658–1.109)
Clinical pregnancy (rate) per transfer	47/182 (25.8)	96/300 (32)	0.807 (0.600–1.085)
Ongoing pregnancy (rate) per transfer	39/182 (21.4)	77/300 (25.7)	0.835 (0.595–1.171)
Implantation rate per transfer	51/293 (17.4)*	104/374 (27.8)***	0.626 (0.465–0.843)
Miscarriage rate per transfer	9/47 (19.1)	27/96 (28.1)	0.681 (0.349–1.329)
Multiple pregnancy rate per transfer	4/47 (8.5)	8/96 (8.3)	1.021 (0.324–3.220)
Ectopic pregnancy rate per transfer	0 (0)	2/110 (1.8)	–
Live birth (rate) per transfer	38/182 (20.9)	67/300 (22.3)	0.935 (0.657–1.330)
Biochemical pregnancy (rate) per woman	54 (44.6)*	93 (60)**	0.744 (0.587–0.942)
Clinical pregnancy (rate) per woman	47 (38.8)*	85 (54,8)**	0.708 (0.543–0.924)
Ongoing pregnancy (rate) per woman	38 (31.4)*	73 (47.1)**	0.667 (0.488–0.911)
Live birth (rate) per woman	38 (31.4)*	67 (43.2)**	0.727 (0.528–1.000)
Time to pregnancy from starting stimulation (months)			
Time to first positive HCG	12.7 (7.3–17.6)*	7.6 (3.8–12.6)***	/
Time to first clinical pregnancy	13.7 (8.2–18.8)*	8 (4.2–13.1)***	/
Time to first ongoing pregnancy	14.7 (9.2–19.8)*	9.5 (5.2–14.3)***	/
Time to first live birth	20.5 (15.4–25.8)*	16 (14;5–22.6)***	/

*Note*: Values are median (25th and 75th percentile) or number (%).

Abbreviations: CI, confidence interval; FET, frozen embryo transfer.

* versus **p < 0.05, * versus ***p < 0.01.

**TABLE 4:** Cox regression analysis of factors for prediction of a cumulative live birth from multiple ovarian stimulation followed by the fresh/frozen transfer cycles in 2 year follow‐up.

Factors	B	SE	Hazard ratio (95% CI)
Age of women**	−1.541	0.025	0.857 (0.815–0.901)
Body mass index	−0.818	0.034	0.982 (0.919–1.050)
Infertility duration	0.023	0.036	1.023 (0.954–1.097)
Primary infertility	0.327	0.226	1.386 (0.890–2.158)
Antral follicle count	0.037	0.067	0.963 (0.845–1.099)
No. of embryos transferred	0.028	0.355	1.029 (0.513–2.062)
Cause of infertility	−0.098	0.088	0.907 (0.763–1.077)
Basal FSH level	−0.027	0.022	0.973 (0.933–1.016)
Basal AMH level	0.109	0.127	1.115 (0.869–1.431)
Embryo banking versus non‐banking strategy*	0.640	0.272	1.896 (1.114–3.229)
No. of transfer cycles	−0.072	0.289	0.931 (0.529–1.639)
Total no. of transferable embryos	0.004	0.073	1.004 (0.869–1.159)
First cycle	−0.163	0.295	0.850 (0.477–1.514)
No. of oocytes per woman	−0.027	0.040	0.973 (0.899–1.053)
No. of stimulation cycles per woman**	−0.0.105	0.030	0.900 (0.849–0.955)

*p < 0.05, **p < 0.01.

**FIGURE 2:**
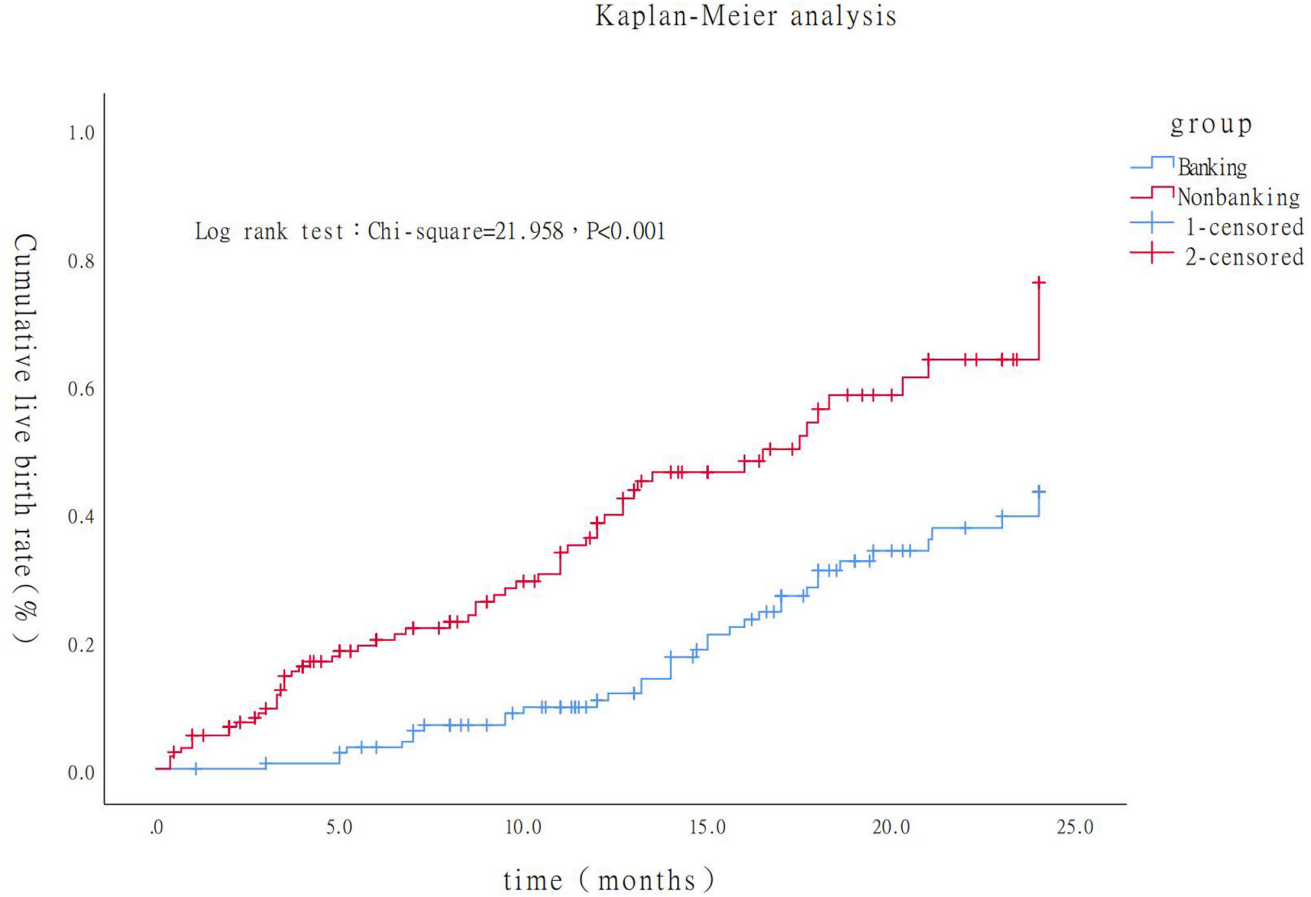
The cumulative incidence of ongoing pregnancy leading to live birth within 24 months followed‐up embryo banking and non‐banking group.

A total of 300 transfer cycles were performed in the non‐banking group including 114 fresh transfer and 186 frozen transfer, whereas 182 frozen transfer cycles were performed in the banking group. Patients in the banking group had undergone 1.5 ± 0.7 transfer cycles, while those in the non‐banking group finished 1.9 ± 0.6 transfer. More women had a single embryo transfer in the non‐banking group compared to that in the banking group. There were no difference in the percentage of day 3 or blastocyst transfer, method of endometrial preparation and the endometrial thickness on the transfer day between the two groups. The biochemical pregnancy, clinical pregnancy, ongoing pregnancy, miscarriage, multiple pregnancy, ectopic pregnancy and live birth rates per transfer were comparable between two groups. However, the implantation rate was significantly lower in the banking group compared to that in the non‐banking group.

The CLBR was significantly lower in the non‐banking group compared to that in the banking group. Moreover, the average time from ovarian stimulation to live birth was significantly longer in the banking group compared to the non‐banking group. In Kaplan–Meier analysis (Figure 2), the cumulative incidence of live birth was significantly lower in the banking group compared to the non‐banking group (Log rank test, chi‐square = 21.958, *p* < 0.001). Medians for survival (live birth) time in non‐banking group was 17.5 (95%CI 12.8–22.2) months, while in the banking group, no more than half of the patient reached a live birth within 24 months follow up.

Cox proportional hazard modeling (Table 4) using the enter method by the age of women, BMI, AFC, duration of infertility, type and causes of infertility, basal FSH, cycle number, embryo pooling strategy, number of stimulation cycles per woman, number of oocytes obtained per woman, and total number of transferable embryos per woman, revealed that the embryo banking, age of women, number of stimulation cycles per woman were strongly associated with the CLBR after adjusting other confounding factors. Particularly, women with no embryo banking was 1.89 times more likely to achieve a live birth compared to those women with embryo banking (HR = 1.896; 95% CI: (1.114–3.229), *p* = 0.018), and number of stimulation cycles per woman was negatively associated with the CLBR (HR = 0.900; 95% CI: (0.849–0.955), *p* < 0.001).

## 4 DISCUSSION

In this retrospective study, we found that embryo banking in women undergoing IVF with POR was associated with a significantly lower cumulative live birth rate and a significantly longer time to live birth when compared with no embryo banking.

The concept of oocyte banking through repeated IVF cycles was first published by Cobo et al.^15^ In that report, oocytes were obtained through multiple stimulation cycles using an antagonist protocol until at least 5 embryos were vitrified for subsequent transfer. They found a significantly higher cumulative live birth rate per women when compared to those who had a single IVF cycle with fresh transfer (36.4% vs. 23.7%). Our embryo banking was similar to that of Cobo et al.^15^ in which all couples were offered several stimulation cycles until at least five embryos were available for transfer. On the other hand, Datta et al.^18^ described the cumulative outcomes of transferring frozen embryos accumulated over three consecutive modified natural cycles (regardless of the number of embryos obtained) in women with low ovarian reserve where the chance of obtaining an embryo is uncertain. The live birth rate following one embryo transfer in this ‘triple freeze program’ was significantly higher than that of one fresh embryo transfer in one modified natural IVF cycle (30.6% vs. 13.3% respectively; *p* = 0.002). Additionally, the cumulative live birth rate including all embryo transfer was also significantly higher in the ‘triple freeze program’. However, the comparison was not fair as it was based on all transfers from embryos banked through three stimulation cycles versus a fresh embryo transfer following one stimulation cycle. In the present study, we included all stimulation cycles in both groups within 24 months after the first stimulation cycle.

Poor outcomes in the treatment of women with POR with a single IVF cycle is usually due to high cycle cancellation rates or lack of good quality embryos for transfer, and there is still no consensus on the optimal stimulation protocols for women with POR.^22^ Similar to our study, Greco et al^16^ selected a mild stimulation protocol with preference to conventional high‐dose protocols for women with POR, in order to make repeated IVF cycles more user‐friendly and thus to prevent women from dropping‐out of treatment. In our study, more women in the banking group adopted PPOS and mild stimulation protocol for ovarian stimulation. PPOS and mild stimulation with clomiphene would lead to elective freezing of all embryos and subsequent frozen embryo transfer.^23^, ^24^

The modern technology of vitrification allows safe cryopreservation of oocytes and embryos with a high post‐warming survival rate. The transfer of frozen embryos in the freeze‐all protocol has been reported to result in improved live birth rates when compared to fresh transfer in women with polycystic ovary syndrome or normal ovarian response.^25^, ^26^, ^27^ However, in the present study we found the implantation rate was inferior in the banking group in which only frozen embryos were replaced. A registry study also showed that a freeze‐all strategy was beneficial in high responders (≥15 oocytes) but not in intermediate (6–14 oocytes) or low (1–5 oocytes) responders.^28^, ^29^ Freezing and thawing may cause damage to embryos especially from women with POR, leading to a lower live birth rate. Majority of women in the non‐banking group used an antagonist protocol and more fresh embryo transfer cycles leading to live birth. This may be a major reason for a short time to live birth in the non‐banking group.

In this study, a similar number of oocytes and transferable embryos per retrieval was noted between the two groups although the two groups used different ovarian stimulation protocols. Strategies with embryo banking up to five instead of fresh transfer could allow more stimulation cycles in the same period. During 24 months follow up, patients in the non‐banking group had undergone 2.6 ± 1.5 stimulation cycles, while those in the banking group finished 5.3 ± 2.8 stimulation cycles. The banking of embryos over multiple cycles would provide a greater number of embryos for selection and transfer of better quality embryos. However, it did not improve the CLBR in women with POR. Cox regression model in our study demonstrated that the number of oocytes obtained, and number of transferable embryos per woman and number of transfer cycles were not associated with the CLBR, but the number of stimulation cycles per woman was negatively associated with the CLBR after adjusting for other confounding factors. It can be argued that multiple retrievals and cryopreservation of embryos in the banking might have incurred in a higher cost compared to that with a single stimulation cycle followed by fresh transfers. And there is an obvious advantage of avoiding further unnecessary stimulation cycles, in case that live birth is achieved in the first or second cycle. Furthermore, women in the banking group were also subject to repeated invasive retrievals procedures, which were associated with a small surgical risk.

The selection criteria in our study were different from those used in previous studies. Cobo et al.^15^ adopted POR criteria described previously by Surrey and Schoolcraft,^30^ whereas Chatziparasidou et al.^14^ included POR according to AFC <7. In contrast, we used POR according to the Bologna criteria,^1^ leading a lower follicle pool and therefore a worse oocyte quality than in other studies. It is difficult to compare our results with previous studies as none of the available studies evaluated the effect of embryo banking on the cumulative live birth rates and the time to live birth. The duration of 24 months follow up in the present study allowed the majority of women to accumulate enough embryos for transfer. CLBR is the outcome of interest for infertile couples and outcomes from several IVF cycles including all subsequent frozen embryo cycles performed within 24 months follow up were evaluated. Other strengths of the present study include none of the patients were lost to follow‐up in the study. Furthermore, we performed a Kaplan–Meier analysis to compare the cumulative live birth rate between the two groups, as it assumed that women who did not return for subsequent FET cycles had the same chance of a pregnancy resulting in a live birth as those who returned for treatment.^31^ Those patients in both groups with remaining frozen embryos and no live birth at the end of the study were regarded as being censored.

The limitations of our study are its retrospective design, a small sample size and being performed at a single center, so results may not be generalizable to other populations and clinical setting. Some imbalance in characteristics were found between two groups and Cox regression analysis was carried out for controlling the bias. Another limitation might be the permitted between‐cycle regimen adjustments in both strategy since patient or physician are apt to try another stimulation protocol if the previous one failed. The number of embryos transferred were different with more women undergoing single embryo transfer in the non‐banking group as some of the patients only had one fresh embryo to transfer after one stimulation cycle. Randomized controlled trials with adequate sample size should be conducted to confirm these findings.

## 5 CONCLUSION

In conclusion, in women with a poor response based on the Bologna criteria starting their IVF/ICSI treatment, embryos banking strategy does not improve cumulative live birth rates while it prolongs time to live birth. Therefore, implement embryos banking strategy in poor responders is not effective compared to conventional non‐banking strategy.

## FUNDING INFORMATION

This study was supported by the Special Fund for Clinical Medical Research of Shanghai Municipal Health Commission (No: 202040127).

## CONFLICT OF INTEREST STATEMENT

The authors declare no conflict of interest.

## HUMAN RIGHTS STATEMENTS AND INFORMED CONSENT

The study protocol was approved by the Institutional Review Board of the hospital (No. KS22357). Informed consent was waived because only unidentified data were used in this retrospective study.
